# The Relationship between Diet Breadth and Geographic Range Size in the Butterfly Subfamily Nymphalinae – A Study of Global Scale

**DOI:** 10.1371/journal.pone.0016057

**Published:** 2011-01-05

**Authors:** Jessica Slove, Niklas Janz

**Affiliations:** Department of Zoology, Stockholm University, Stockholm, Sweden; University of Plymouth, United Kingdom

## Abstract

The “oscillation hypothesis” has been proposed as a general explanation for the exceptional diversification of herbivorous insect species. The hypothesis states that speciation rates are elevated through repeated correlated changes – oscillations – in degree of host plant specificity and geographic range. The aim of this study is to test one of the predictions from the oscillation hypothesis: a positive correlation between diet breadth (number of host plants used) and geographic range size, using the globally distributed butterfly subfamily Nymphalinae. Data on diet breadth and global geographic range were collected for 182 Nymphalinae butterflies species and the size of the geographic range was measured using a GIS. We tested both diet breadth and geographic range size for phylogenetic signal to see if species are independent of each other with respect to these characters. As this test gave inconclusive results, data was analysed both using cross-species comparisons and taking phylogeny into account using generalised estimating equations as applied in the APE package in R. Irrespective of which method was used, we found a significant positive correlation between diet breadth and geographic range size. These results are consistent for two different measures of diet breadth and removal of outliers. We conclude that the global range sizes of Nymphalinae butterflies are correlated to diet breadth. That is, butterflies that feed on a large number of host plants tend to have larger geographic ranges than do butterflies that feed on fewer plants. These results lend support for an important step in the oscillation hypothesis of plant-driven diversification, in that it can provide the necessary fuel for future population fragmentation and speciation.

## Introduction

Herbivorous insects constitute about a quarter of all living species [1], and butterflies make up an important part of that diversity. The greater species diversity of herbivorous insect groups, as compared to their non-plant-feeding sister-groups [2], [3], suggests that host use may be relevant to explaining insect diversity [but see 4]. Previously, studies have focused mainly on host plant specialisation. But specialisation is a depletive source of diversification and would run out of variation to act upon – preventing further specialisation – and would then run into a dead end [5]. Yet specialisation is not a dead end. Rather diet breadth is a dynamic trait with evidence of shifts and expansions as well as specialisation [6]–[8]. And these changes in host use may be the necessary injection of new variation that facilitates diversification [5], [9].

The “oscillation hypothesis” [5], [10] proposes that the increased diversity of herbivorous insects is largely a result of expansions in diet breadth followed by specialisation, in other words oscillations in diet breadth. These oscillations are then coupled with correlated changes in geographic range size, which in turn may lead to population fragmentation.

An important requirement for the oscillation hypothesis is that diet breadth should be correlated with geographic range size, as wide geographic ranges will set the stage for subsequent local adaptation and specialisation. This is because in a larger geographic range the environment is likely to be more heterogeneous, with differences in, for example, climate, local abundance of host plants, or interactions with competitors or parasites. Although gene flow can be high during periods of expansion, it may decrease with time as populations become increasingly adapted to local conditions. This causes the oscillation to swing back toward a more specialised use of locally favoured host plants, and this geographic variation in host use may give rise to population fragmentation and speciation. That is, expansions in diet breadth and geographic range are the source of new variation that allows further specialisation and speciation, and hence may be an important process behind the diversification of plant-feeding insects. There is support for this in the increased diversification of insect groups that have passed through such an oscillation in diet breadth as compared to their primitively specialised sister-groups [5], [11]. The greater diversity of these groups corresponds to the predictions made by the oscillation hypothesis, but the mechanistic assumptions underlying the process remain to be tested, in particular whether increased diet breadth is positively correlated with geographic range.

Previous studies considering the relationship between diet breadth and geographic range have found a positive correlation [12]–[15]. However, the studies have all compared range sizes within a very restricted area, e.g., Germany and the United Kingdom. Range sizes of butterflies and other species do not follow national borders, and hence it is very likely that the ranges of some – if not most – species extend quite a bit outside the region under study. This means that “range size” does in fact not measure range size, but rather the ability of the species to persist within the various types of habitat that this particular region offers. As a consequence, it is important to perform the study on a geographic level that includes the whole geographic ranges of all included species. This problem will persist in any geographic region, no matter its size, and the only way to avoid it is to perform the study on a global level. This is to our knowledge the first study that investigates this relationship between diet breadth and geographic range size with a global scope. Butterflies are among the few groups where comprehensive data is available on this scale, much due to long-standing and widespread attention the group has received from amateurs, collectors and researchers.

The aim of this paper is to test if there is a correlation between the diet breadth and geographic range size in the butterfly subfamily Nymphalinae at a global scale. The Nymphalinae is very suitable for this study, as it is a very diverse group with variation in both diet breadth and geographic range sizes. Moreover, the recent development of well-supported phylogenies allows diet breadth and geographic range to be analysed phylogenetically.

## Results

The maximum likelihood analyses resulted in a well-supported phylogeny (Figure S1), which is largely consistent with those previously published for Nymphalinae [16]. The decision whether or not to account for the phylogeny was based on the results from the test of phylogenetic signal, which tests if species are independent of each other with respect to the variables studied, in this case, diet breadth and geographic range. Both diet breadth measures received intermediate lambda estimates (0.48 for diet breadth index and 0.45 for number of genera), indicating that there was some effect of phylogeny. Geographic range on the other hand appeared to be weakly correlated to phylogeny (λ = 0.08). As geographic range and diet breadth gave such different results, and because the intermediate value for diet breadth was difficult to interpret, we chose to analyse the data using both cross-species comparisons and accounting for phylogeny using GEE.

**Figure 1:**
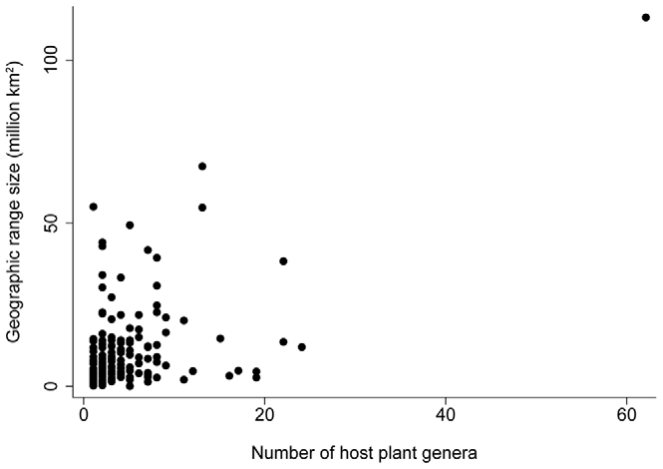
The relationship between geographic range size and the number of host plant genera. 182 Nymphalinae species are included and the outlier is Vanessa cardui. The data for range size and host diversity are both highly skewed, with most species having small ranges and/or feeding on a few host plants. (Plotting the index instead of number of genera produces a similar relationship.) To correct for this skew, data was box-cox transformed for the cross-species comparison (Figure 2) and analysed phylogenetically using Generalised Estimating Equations, which allows non-normal response variables.

**Figure 2:**
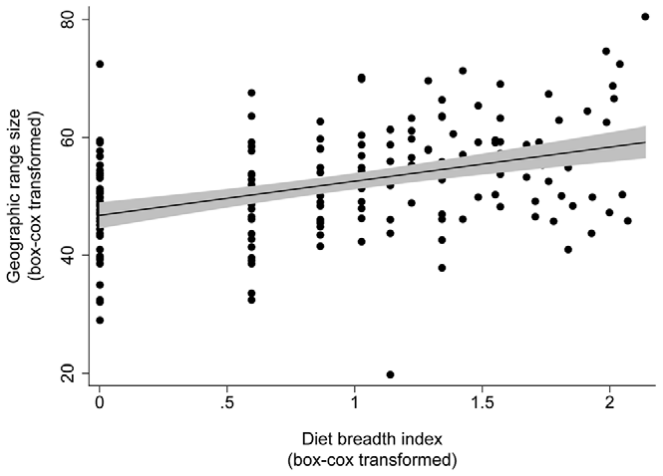
Results for the cross-species comparison. Linear regression of geographic range size on the diet breadth index (number of host plant genera multiplied by the number of families and orders). The black line is the regression line (fitted values) and the grey area is the 95% confidence interval. Both traits are box-cox transformed to correct for the skewed distribution of data (Figure 1). (Data was also analysed using Generalised Estimating Equations to account for phylogeny.)

Cross-species comparisons were performed using linear regression. However, because both diet breadth and geographic range data were strongly right-skewed, that is, most species have very small diet breadths and geographic ranges (Figure 1) [17], data had to be transformed before analysis. Box-Cox transformations gave approximate normal frequency distribution for both traits. Linear regression showed that geographic range is significantly correlated with diet breadth (Figure 2, diet breadth index: df = 181, r^2^ = 0.17, p<0.001; number of genera: df = 181, r^2^ = 0.15, p<0.001).

A significant correlation between geographic range and diet breadth was also found when accounting for phylogenetic non-independence using GEE (diet breadth index: df_phylogenetic_ = 39.74, estimate  = 3.1×10^−8^, p = <0.001; number of genera: df_phylogenetic_ = 39.74, estimate  = 1.9×10^−8^, p = <0.001). This result is consistent for removal of the outlier *Vanessa cardui* (Figure 1).

## Discussion

Geographic range size was found to be significantly correlated with diet breadth in Nymphalinae on a global scale. This suggests that butterflies that have a more diverse host plant use are more geographically widespread than butterflies with more narrow host plant use. These results agree with previous studies showing that geographic range is correlated to diet breadth [12]–[14]. However, Brändle *et al.* [14] showed that the strength of this correlation decreased with increasing scale. The global perspective employed in this study ensures that the entire ranges of the species are considered, and the results show that the correlation is present at this global scale. Diet breadth explains 17% of the total variation in geographic range size. Considering the multitude of other factors that would be expected to influence a species' geographic range (such as climate, competition, and habitat availability), this must be seen as rather high.

In order to evaluate whether closely related butterflies are more similar than expected by chance with respect to diet breadth and geographic range, we tested both traits for phylogenetic signal. The results showed that, although diet breadth does show some effect of phylogeny, it is not highly conservative. This is interesting considering that the particular host plant taxa used *are* conservative, with related butterflies feeding on related plants [18], [19]. Most host shifts are to ancestrally used host plants [8], [19], which suggests that using a completely new host is relatively difficult, whereas adding and subtracting from a potential range of ancestrally used host plants is easier. In other words, host use is conservative with few drastic changes in the host plant species used, but at the same time dynamic with relatively common changes in diet breadth [20]. Geographic range, on the other hand showed little correlation to phylogeny. Results from previous studies are contradictory [12], [21]–[24], and the use of different methods makes the results hard to compare. Although more phylogenetic studies are necessary, at the moment evidence appears to favour geographic range size as relatively independent of phylogeny [25]. Because the results for diet breadth and geographic range differed in their phylogenetic signal, both cross-species comparisons and GEE with phylogeny incorporated were used to test for correlation between the two traits.

Irrespective of which method was used, butterflies with larger diet breadths had significantly larger geographic ranges. This lends support for an important part of the oscillation hypothesis in that it can provide the necessary raw material for forthcoming population fragmentation and speciation [10]. However, the results do not imply anything about the causation.

The original reasoning in the formulation of the oscillation hypothesis was that expansions in diet breadth should precede expansions in geographic range [10]. Ultimately, the geographic range of the host plant(s) used sets the limit of the geographic range of the herbivore, and as two plant taxa rarely have fully overlapping ranges the potential range after a host plant colonisation should be larger than before the event. Hence, an increase in diet breadth allows the insect to expand its geographic range. However, the opposite is also possible: an expansion of the geographic range could put the herbivore in contact with novel plant species and thus increase the likelihood of colonisation through ecological fitting [26]. This alternative implies that the insect's initial geographic range was constrained by something else than the geographic range of its host, and that a change in some external factor caused its geographic range to expand. It is a scenario that is reminiscent of the “taxon pulses” proposed by Erwin [27], [28] to explain the geographic expansion and subsequent turnover of clades through time [also see 29], [30]. While this scenario requires a secondary explanation for the geographic expansions (such as climatic change) it could well lead to the same correlation between diet breadth and geographic range. Hence, while we have demonstrated the correlation predicted by the oscillation hypothesis, we cannot yet distinguish between the alternative scenarios that may have given rise to the pattern. However, it must also be pointed out that the two scenarios are not mutually exclusive and it may well be that they are actually two sides of the same coin.

As mentioned, incorporating a novel host into the repertoire is not a trivial task, and is likely to require some pre-existing machinery that allows at least some individuals of the insect species to have some realized fitness on the novel plant at the time of colonisation [26], [29], [ also see 31], [32]. In other words, even in the case when a host plant colonisation appears to be caused by a geographic range expansion, some ability to utilize this particular host must have existed in the species prior to the expansion.

The oscillation hypothesis was proposed as a general explanation for the elevated diversification rates in plant-feeding clades. Previous studies have found support for the overall pattern expected from the hypothesis, that diversity in host plant use is correlated with increased diversification rates [5], [11]. However, the hypothesis is dependent upon several distinct steps, each of which requires to be demonstrated separately. There is previous evidence for the first step, that there are at least transient phases of expansion in diet breadth, i.e. specialisation is not a dead end [6]–[8]. There is also evidence for the last step, that a large geographic range may increase the likelihood of fragmentation, isolation and speciation [e.g. 33]. Plausibly, a larger geographic range will encompass more environmental heterogeneity and a more pronounced geographic mosaic [33], [34]. Such spatial variation may often lead to divergent selection, reproductive isolation and speciation, and in some cases it may be as a direct result of different interactions leading to diversifying coevolution [33]–[35]. In addition, an increase in geographic range per se, without divergent selection pressures, can lead to genetic differentiation with effects similar to local adaptation [36].

The results presented here support the remaining middle step where a diet breadth expansion leads to a correlated increase in geographic range. As a consequence, the general plausibility of the oscillation hypothesis as a driver of diversification has been strengthened. It remains to be seen, however, to what extent these results can be extrapolated to other butterflies, and to other insect groups with different patterns of geographic distribution and different feeding habits.

We conclude that the geographic range sizes of Nymphalinae butterflies are correlated with diet breadth on a global scale. In other words, butterflies that have a broader diet breadth in general have larger geographic ranges than do butterflies with more limited diet breadths. Such large geographic ranges could increase the likelihood of future fragmentation and speciation, as a result of diversifying coevolution. Our study provides evidence for an important mechanism behind the oscillation hypothesis for the diversification of plant feeding insects, where an expansion in diet breadth is correlated to an expansion in geographic range.

## Methods

Nymphalinae is a diverse subfamily containing approximately 496 species in 56 genera [16]. For this study, data was available for 182 species in 36 genera, which covers all five tribes. The phylogenetic analyses were performed on a subset of 144 species (in 35 genera) for which sequence data are available from previous studies [16], [37]–[44]. Hence, although we used all species for which data were available, the data set still represents an incomplete sample. However, there is no apparent reason to expect sampling bias that might affect the results.

### Phylogeny

Sequence data from three gene regions were included, COI (Cytochrome Oxidase subunit I, EF-1α (Elongation Factor 1 alpha) and wingless, these are available on Genbank (for accession numbers see Table S1). Sequences were downloaded and aligned in BioEdit v7.0.5.3 [45]. The alignment was straightforward.

The fit of different nucleotide substitution models were estimated based on likelihood scores calculated in PAUP* 4.0 [46] analysed on the ModelTest server 1.0 [47] running ModelTest 3.8 [48] using the standard AIC (Akaike Information Criterion) and using branch lengths as parameters. The suggested model, GTR+I+G, was imposed on the three gene partitions separately and Maximum likelihood (ML) analysis was conducted using RAxML 7.0.4 [49] via the Cyberinfrastructure for Phylogenetic Research (Cipres) Portal v.1.15 [50] using the default parameters. Bootstrap values were calculated from 1000 pseudoreplicates.

### Diet breadth and geographic range data

Host plant data was collected from several sources [8], [51]–[63]. Because host plant data may contain spurious/anecdotal records, a number of measures were taken to ensure a consistent approach to which records were included. We recorded the plant genera used, as records at species level may be uncertain or lacking. Records of observations made in the lab were not included as these may represent the butterfly's potential host use, rather than actual host use, and plants that are not used in the wild will not contribute to the geographic range of the butterfly (although they may lead to expansion and future increase in geographic range). In addition, rearings in the lab are lacking for many butterfly species and host plants. Further, we employed the same steps as used previously by Janz & Nylin [19] and Janz *et al.* [5], where records are only included if at least one of the following criteria is met: 1) several plant genera are reported in a family, 2) several species are reported in a plant genus, 3) the plant genus is used by other butterflies in the genus, or 4) there are several independent sources. Where species and subspecies status differed between host plant data and data on geographic range, we followed the nomenclature used for the geographic range data. Two measures of diet breadth were used (Table S2). The first measure was the number of genera used. The second measure was designed to reflect the greater diversity in diet breadth of butterflies that feed on plants not only in several genera but also several families and even orders. For this purpose, the plant genera were assigned to family and order according to Stevens [64] and the number of families and orders were then multiplied with the number of genera to create an index of diet breadth. The delimitation of genera and families may not be directly comparable across plant groups, which may add to the noise in the host range data-set. However, we still believe that these two measures of host range will reflect the degree of polyphagy of the butterflies and that using more refined measures of host range would only strengthen any patterns found.

Geographic range data was collected from Savela [62] as automatically digitised maps. The accuracy of these automatically generated range maps was checked against available range maps [51], [60], [63] and adjusted if necessary. The downloaded maps were in a map projection that was not of equal area, therefore the maps were recreated in a GIS (ArcMap 9.2) to avoid latitudinal distortion. The downloaded maps were used to create selections on the world map that is distributed with the software and using the statistics tool, the area in km^2^, was obtained for each species geographic range (Table S2).

### Analyses

To test if species are independent of each other with respect to diet breadth and geographic range, lambda was estimated using BayesTraits v1.0 [65], implementing BayesContinuous [66] and Maximum Likelihood. Lambda measures if phylogeny correctly predicts the patterns of covariance among species on a given trait, that is, the strength of the phylogenetic signal. Lambda was assessed for geographic range and both measures of diet breadth.

Correlation between diet breadth and geographic range was analysed using cross-species comparison, under the assumption that the traits are independent of history. As both diet breadth and geographic range data were strongly right skewed (Figure 1) they were modified by Box-Cox transformation (transformation parameters: t_geographic range_ = 0.132, t_diet breadth index_ = −0.459, t_number of genera_ = −0.349). Linear regression was performed in Stata/SE (v. 11.0).

If instead related species are assumed to be more similar than expected by chance, the analysis needs to take phylogenetic relationships among species into account. This was done using generalised estimating equations (GEE), which incorporates species relatedness as a correlation matrix and uses a generalised linear model approach, which allows data to be analysed using non-normal response variables. The data (highly skewed, positive and continuous) suggested using the gamma family and log link. Branch lengths for the phylogeny were proportional to the number of changes along each branch. The GEE analyses were run using the APE package [67], [68] in R 2.9.2 [69].

## Supporting Information

Figure S1 **Phylogeny of Nymphalinae reconstructed using maximum likelihood analysis.** Numbers beside internal nodes are maximum likelihood bootstrap values obtained from RaxML.
(TIF)

Table S1 **List of taxa used in this study with their Genbank accession numbers.**
(XLSX)

Table S2 **Diet breadth and geographic range data for 182 Nymphalinae species.** Data used in the analyses. Diet breadth measures used in the analyses were number of genera and diet breath index. Diet breadth index was calculated by multiplying the number of host plant genera by the number of families and orders used.
(XLSX)
