# Supplementary figures and images for: The Relationship between Diet Breadth and Geographic Range Size in the Butterfly Subfamily Nymphalinae – A Study of Global Scale
Source: PLoS One. 2011 Jan 5;6(1):e16057. doi: 10.1371/journal.pone.0016057 (PMC3016415; doi:10.1371/journal.pone.0016057)

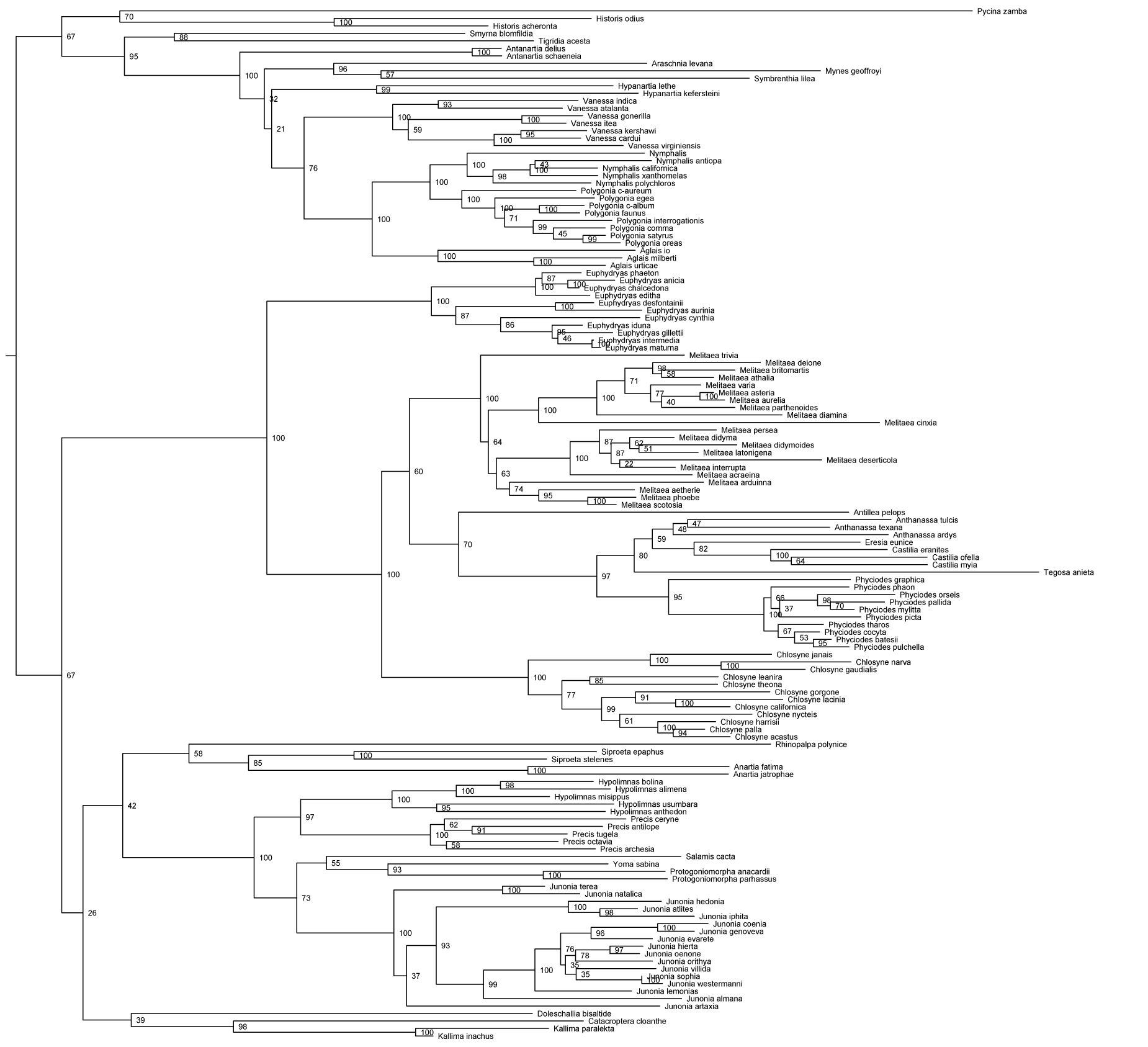

Supplement: Figure S1 — Phylogeny of Nymphalinae reconstructed using maximum likelihood analysis. Numbers beside internal nodes are maximum likelihood bootstrap values obtained from RaxML. (TIF) [file pone.0016057.s001.tif]
